# Officers

**Published:** 1867-07

**Authors:** 


Dr. C. Goodbrake, Chairman of the Nominating Committee submitted the following report:—

*For President*—Dr. S. W. NOBLE, of Bloomington.

*For Vice-Presidents*—Drs. D. W. YOUNG, of Aurora, and O. Q. HERRICK, of Kansas.

*For Treasurer*—Dr. J. H. HOLLISTER, of Chicago.

*For Next Place of Meeting*—Quincy.

On motion of Dr. H. A. Johnson, the report of the Committee was accepted, and the several candidates for office unanimously elected.

The President appointed Drs. Johnson, Miller, and Moore a committee to conduct the newly-elected officers to their places.

Drs. Noble and Young, on being conducted to their places, thanked the Society for the honor conferred on them; and the retiring President, Dr. Haller, delivered a short, but appropriate address.

The annual assessment for the year 1867, was fixed at $3.

Dr. H. A. Johnson then moved a reconsideration of the vote adopting the constitutional amendment relating to voting by permanent members.

The motion was carried, and, after a brief discussion by Drs. Johnson, Watson, Moore, Niles, Young, Worrell, and Davis, the amendment was again adopted, by a unanimous vote of 49 ayes, 0 nays.

On motion, the Society adjourned to 2 o'clock P.M.

AFTERNOON SESSION.

The Society was called to order at 2 o'clock P.M., Dr. S. W. Noble, President, in the chair.

Dr. J. Robbins, of Quincy, moved to amend the minutes of the last annual meeting, by inserting the following in the place of the fifth paragraph from the bottom of page 5 of the printed transactions:—

"A paper was presented from the Adams County Medical Society, remonstrating against the admission of the Quincy Medical Society to representation in this Society, which was referred to a special committee, consisting of S. T. Trowbridge, H. Noble, and J. M. Steele.

"The special committee, to whom was referred the communication from the Adams County Medical Society, reported the following resolution, which was adopted:—

"*Resolved*, That the so-called Quincy Medical Society is not entitled to representation in the Illinois State Medical Society."

After some discussion, the amendment proposed by Dr. Robbins was adopted, and the minutes, as amended, approved.

The special order of business being the report of the Standing Committee on Practical Medicine, Dr. J. A. Allen, Chairman of that Committee, read a paper on the Prevalence of Cholera in Chicago during the year 1866, written by Dr. W. R. Marsh of Chicago. The report was founded almost exclusively on the records of the Health-Officer of Chicago, and after some discussion by Drs. N. S. Davis, H. A. Johnson, and D. Prince, it was, on motion of Dr. P. H. Bailhache, referred to the Committee of Publication.

An invitation was received from the Mayor and city authorities of Springfield, to visit Oak Ridge Cemetery at 4 o'clock P.M., which was accepted.

Dr. J. Adams Allen presented the following resolution:—

*Resolved*, That the moderate use of ripe, but not stale or decaying, fruits is not objectionable as tending to produce cholera, but, rather, conducive to the preservation of health during the hot season.

This elicited a discussion, which was participated in by Drs. Edgar, Allen, Johnson, and Prince. While the latter was speaking, the hour fixed for the excursion to Oak Ridge arrived, and the further consideration of the subject was postponed until 9 o'clock Wednesday morning.

Notice was given that a public lecture would be given in the Hall, by Dr. J. Adams Allen, of Chicago, at 8 o'clock that evening.

The Society then adjourned to 9 o'clock A.M. of Wednesday.

WEDNESDAY, JUNE 5TH.

The Society was called to order at 9 o'clock A.M., Dr. S. W. Noble, President, in the chair.

Dr. N. Wright, in behalf of the Committee of Arrangements, reported the following list of volunteer communications, with a recommendation that they be heard in order after the reports of committees.

Tracings of the Pulse, and Shygmograph for making the same, prepared by H. A. Johnson, M.D., Prof. Chicago Med. College.

Experimental Inquiries concerning the Physiological Effects of Alcoholic Drinks. By N. S. Davis, M.D., Prof. Chicago Med. College.

Treatment of Paniform Cornea, occurring with Granular Eye-Lids. By Joseph S. Hildreth, M.D., Chicago.

Pocket Obstetric Forceps, with a description of the same. By Addison Niles, M.D., of Quincy.

A Case of Hydrophobia, with Treatment. By T. R. Higgins, M.D., of Vandalia.

Deformities of the Spine treated by Mechanical Appliances. By F. O. Earle, M.D., of Chicago.

Dr. J. H. Hollister offered the two following resolutions, which were unanimously adopted.—

*Resolved*, That the Treasurer report to the Publishing Committee, at the close of each annual meeting, the names of all those members who have been present at any one or more of the annual meetings during the *five* preceding years; or who have paid one or more annual assessments during that time; or have reported physical disability, preventing attendance at said meetings. And that only the names so reported be published in the Transactions for that year.

*Resolved*, That the Treasurer be instructed to communicate each year with all the members whose names appear in the Transactions, who have not paid their annual dues, and solicit payment of the same.

Dr. David Prince offered the following, which was adopted unanimously:—

*Resolved*, That it is with deep regret that the members of this Society contemplate the death of Daniel Brainard, M.D., Professor of Surgery in Rush Medical College; and that we unanimously accord to his memory the high appreciation due to the name of one who, by his talents and industry, has added to the sum of human knowledge, thus placing the world, as well as the profession, under obligations to perpetuate the memory of his contributions.

Dr. T. F. Worrell moved that a committee of five be appointed as a judicial committee to investigate the charges against Dr. Addison Niles, of Quincy. The motion was adopted, and the President appointed Drs. David Prince, E. W. Moore, DeLaskie Miller, O. Q. Herrick, and L. T. Hewins as said committee. And at a subsequent stage of the proceedings, the names of Drs. H. A. Johnson and Edwin Powell were added to the committee.

Dr. F. B. Haller. of Vandalia, offered the following resolution:—

*Resolved*, That we, the members of the State Medical Society, recommend that the several medical schools in this State adopt the plan of teaching recommended by the recent teachers' convention, in Cincinnati, as soon as practicable; and that we will send our pupils to the school or schools adopting such plan.

Dr. J. Adams Allen moved that the consideration of the subject be postponed until the reports from standing and special committees had been received and disposed of. Which motion was adopted.

